# Age-Related Differences in Naturally Acquired T Cell Memory to *Plasmodium falciparum* Merozoite Surface Protein 1

**DOI:** 10.1371/journal.pone.0024852

**Published:** 2011-09-16

**Authors:** Kiprotich Chelimo, Paula B. Embury, Peter Odada Sumba, John Vulule, Ayub V. Ofulla, Carole Long, James W. Kazura, Ann M. Moormann

**Affiliations:** 1 Center for Global Health Research, Kenya Medical Research Institute, Kisumu, Kenya; 2 Center for Global Health and Diseases, Case Western Reserve University, Cleveland, Ohio, United States of America; 3 Maseno University, Maseno, Kenya; 4 Laboratory of Malaria and Vector Research, National Institute of Allergy and Infectious Diseases, National Institutes of Health, Rockville, Maryland, United States of America; 5 Department of Pediatrics and Department of Quantitative Health Sciences, University of Massachusetts Medical School, Worcester, Massachusetts, United States of America; University of Copenhagen, Denmark

## Abstract

Naturally acquired immunity to *Plasmodium falciparum* malaria in malaria holoendemic areas is characterized by the gradual, age-related development of protection against high-density parasitemia and clinical malaria. Animal studies, and less commonly, observations of humans with malaria, suggest that T-cell responses are important in the development and maintenance of this immunity, which is mediated primarily by antibodies that slow repeated cycles of merozoites through erythrocytes. To advance our rather limited knowledge on human T-cell immunity to blood stage malaria infection, we evaluated CD4 and CD8 T-cell effector memory subset responses to the 42 kDa C-terminal fragment of Merozoite Surface Protein 1 (MSP1_42_), a malaria vaccine candidate, by 49 healthy 0.5 to ≥18 year old residents of a holoendemic area in western Kenya. The proportion of individuals with peripheral blood mononuclear cell MSP1_42_ driven IFN-γ ELISPOT responses increased from 20% (2/20) among 0.5–1 year old children to 90% (9/10) of adults ≥18 years (*P* = 0.01); parallel increases in the magnitude of IFN-γ responses were observed across all age groups (0.5, 1, 2, 5 and ≥18 years, *P* = 0.001). Less than 1% of total CD4 and CD8 T-cells from both children and adults produced IFN-γ in response to MSP1_42_. However, adults had higher proportions of MSP1_42_ driven IFN-γ secreting CD4 and CD8 effector memory (CD45RA^−^ CD62L^−^) T-cells than children (CD4: 50.9% vs. 28.8%, *P* = 0.036; CD8: 52.1% vs. 18.3%, respectively *P* = 0.009). In contrast, MSP1_42_ driven IFN-γ secreting naïve-like, transitional (CD45RA^+^ CD62L^+^) CD4 and CD8 cells were the predominant T-cell subset among children with significantly fewer of these cells in adults (CD4: 34.9% vs. 5.1%, *P* = 0.002; CD8: 47.0% vs. 20.5%, respectively, *P* = 0.030). These data support the concept that meaningful age-related differences exist in the quality of T-cell immunity to malaria antigens such as MSP1.

## Introduction

Malaria is a global health problem that affects primarily infants and children less than 5 years old [1], 2, whereas older children and adults in most endemic regions develop naturally acquired immunity that protects against high-density parasitemia and malaria morbidity [3], [4]. It is clear that antibodies directed against blood stage parasites are critical to this protection since passive transfer of Ig isolated from sera of immune African adults to children with acute malaria rapidly reduces the level of asexual parasitemia and severity of malaria illness [5]. Unlike the clear-cut importance of antibodies in mediating naturally acquired immunity, the role of T-cell memory in the development and maintenance of this immunity is poorly understood. Studies of rodent malaria models and limited observations of malaria-naïve humans who became resistant to blood stage *P. falciparum* challenge after repeated inoculation and drug cure with a small number (∼300) of infected erythrocytes suggest that T-cells and IFN-γ responses, even in the absence of antibodies, confers a degree of protective immunity [6], [7]; however it is unclear whether residual anti-malarial drugs may have contributed to the protection seen [8]. In addition, due to the technical challenges of conducting more elaborate T-cell studies, limited information is available on human memory T-cells particularly in response to defined blood-stage malaria antigens. Greater understanding of how malaria specific T-cell memory subsets contribute to immunity in malaria endemic populations is important to the design and testing of blood stage malaria vaccines as well as understanding how decreasing malaria exposure due to vector control in Africa and elsewhere may affect age-related susceptibility to malaria infection and clinical illness.

Merozoite Surface Protein 1 (MSP1) is one of the most abundant antigenic proteins expressed by asexual parasites of all malaria species. In the case of *P. falciparum*, MSP1 is a 200 kDa glycoprotein expressed and sequentially processed to yield a 42 kDa (MSP1_42_) fragment, which is essential to the initial low affinity attachment of the merozoite to the erythrocyte surface [9]. T-cell epitopes recognized by humans with *P. falciparum* infection are contained within MSP1_33_ sub-fragment that is shed from MSP1_42_ before erythrocyte invasion [10], [XPATH ERROR: unknown variable "rids-text".]. Although the mechanisms by which CD4 T-cells contribute to protective immunity are not well understood, it is likely that this occurs through cytokines that provide help to antigen specific B-cells, e.g. Ig isotype and IgG subclass switching and/or by direct cellular communication with macrophages, CD8 T-cells and B-cells [12], [13], [14], [15]. MSP1-driven IFN-γ responses have been observed in T cell receptor transgenic mice that resolved *P. chabaudi chabaudi* by generating T-cell responses to MSP1_33_, which augment antibody responses to MSP1_19_ [16] and through induction of IL-4 [17]. Vaccination of rhesus monkeys with recombinant MSP1_42_ [18], [19] and human vaccine trials with MSP1 [20], MSP1_19_ [21], and MSP1_42_ [20], [21], [22] lend further support to the role of T-cells in protective immunity. In essence, depletion of IFN-γ and CD4 T-cells abrogates protective immunity in mice immunized with MSP1 [23].

Evaluation of effector memory T-cell subsets in malaria exposed human populations has been constrained by the complexity of the assays involved in the identification of low frequency antigen-specific T-cell subsets, the limited number of peripheral blood lymphocytes that can be obtained during field studies (particularly from infants and children), and the inability to access primary lymphoid organs. However with recent technologic advances, human T-cell memory subsets can be defined by multi-parameter flow cytometry using a panel of functional and phenotypic markers [24]. To this end, CD4 and CD8 T-cell central memory (T_CM_), effector memory (T_EM_), terminally differentiated RA re-expressing effector memory (T_EMRA_) and naïve (T_N_) cell subsets can be characterized according to expression of CD45RA, CCR7, and CD62. T_CM_ are CD45RA^−^CCR7^+^CD62L^+^; T_EM_ are CD45RA^−^CCR7^−^ CD62L^−^; T_EMRA_ are CD45RA^+^ CCR7^−^CD62L^−^; and T_N_ are CD45RA^+^ CCR7^+^ CD62L^+^ [25]. One recent study used a similar approach to characterize T-cell memory subsets specific to *P. falciparum* MSP1_42_ from malaria-naïve adult volunteers who participated in a phase I vaccine trial. This study reported that memory CD4^+^CD45R0^+^CD154^+^ cells were elicited after vaccination [22]. Further, multifunctional cytokine secreting T-cell subsets specific to *P. falciparum* Apical Membrane Antigen 1 (AMA1) have been defined in malaria-naïve individuals vaccinated with this antigenic protein [26]. However, to our knowledge, there are no published data defining MSP1-specific memory T-cell subsets in populations naturally exposed to *P. falciparum*. In the present study, we characterized the frequency and quality of naturally acquired IFN-γ producing MSP1_42_ specific CD4 and CD8 T-cell effector memory subsets in children and adults residing in a malaria holoendemic area of western Kenya.

## Results

### Malaria infection status, white blood cell counts and T lymphocyte subsets of study participants

Table 1 describes the age-stratified median malaria parasite density, complete blood count (i.e. white blood cells, (WBC), total lymphocytes, monocytes, granulocytes, red blood cells (RBC), and platelets), CD4 and CD8 cell absolute counts and frequencies of the study participants. The upper range of asexual parasitemia among these healthy malaria asymptomatic individuals was highest among participants who were 1 to 5 years old compared to infants with a median age of 0.5 years, presumably due to protective maternally acquired antibodies during infancy and the development of malaria-specific immunity later in life. None of the adults were parasitemic by blood smear. Median WBC, lymphocyte, and monocyte counts determined by Coulter Counter were significantly lower among the adults than 0.5 to 5 year olds (*P*<0.001). However, the relative proportion of CD4 and CD8 cells was not different across the various age groups (*P* = 0.310). These latter data are similar to observations of non-African children showing that the absolute number, but not ratio of CD4 and CD8 cells, change normally between birth and approximately 5 years [27], [28].

**Table 1:** Parasitemia, MSP1-specific IgG antibody responses and age-related hematologic indices.

Age in years (sample size)	0.5 (n = 17)	1 (n = 16)	2 (n = 19)	5 (n = 18)	>18 (n = 18)	*P*-value
*P. falciparum* parasite density	0 (0–80)	80 (0–1880)	160 (0–6800)	80 (0–3650)	0 (0–0)	0.071
MSP1_42_-IgG antibody titers (AU)	0.635 (0.228–2.61)	1.84 (0.755–3.13)	1.14 (0.85–4.28)	2.47 (0.94–8.71)	4.71 (1.48–33.0)	0.048
White blood cells	12600 (8800–13500)	13200 (8950–18000)	11100 (8800–13100)	8850 (6525–10250)	5550 (4800–6875)	<0.001
Lymphocytes	7700 (6100–8500)	8400 (5300–10500)	7500 (5600–8950)	4950 (3500–6300)	2700 (2225–3475)	<0.001
Monocytes	1000 (700–1300)	1100 (650–1900)	800 (700–1200)	700 (400–800)	400 (400–500)	<0.001
Granulocytes	3400 (2100–3900)	4100 (2450–5250)	2900 (2050–3650)	3250 (1800–4050)	2450 (1825–2775)	0.063
RBC	4460000 (3750000–5020000)	4730000 (4000000–4845000)	4140000 (3990000–4645000)	4225000 (3983000–4560000)	4390000 (3918000–5015000)	0.469
Platelets	388000 (265000–434000)	344000 (248500–500000)	323000 (217000–464500)	263500 (200250–339750)	223000 (140250–278000)	0.011
**CD4 T cells**						
Absolute counts	2788 (1966–3943)	2397 (2132–3960)	2656 (2228–3348)	2413 (1644–2837)	1576 (669.4–1883)	0.006
Frequencies (%)	37.21 (30.17–41.98)	41.65 (33.95–46.90)	31.75 (29.94–48.91)	45.73 (41.99–48.48)	40.32 (36.64–41.35)	0.163
**CD8 T cells**						
Absolute counts	910 (858–1354)	1214 (893–1890)	1456 (1312–1696)	887 (551–1332)	741 (592–1097)	0.0112
Frequencies (%)	14.84 (14.31–20.98)	15.70 (10.38–21.10)	18.34 (14.69–20.28)	17.36 (14.52–19.67)	19.32 (17.20–22.45)	0.347
CD4∶CD8 Ratio	2.75 (1.9–3.65)	2.1 (1.5–2.85)	1.65 (1.53–2.85)	2.2 (1.3–3.05)	1.85 (0.78–2.65)	0.310

Parasitemia and hematologic index data are expressed as median values per µL of blood with 25th and 75th percentiles.

IgG titers are expressed in arbitrary units (AU).

Optical density values (IgG) and median absolute counts of all hematologic indices decreased with an increase in age (*P*<0.05, Kruskall-Wallis test) except for red blood cells (RBCs) and granulocytes.

### PBMC IFN-γ responses to MSP1_42_ increase after age 5 years

**Figure 1:**
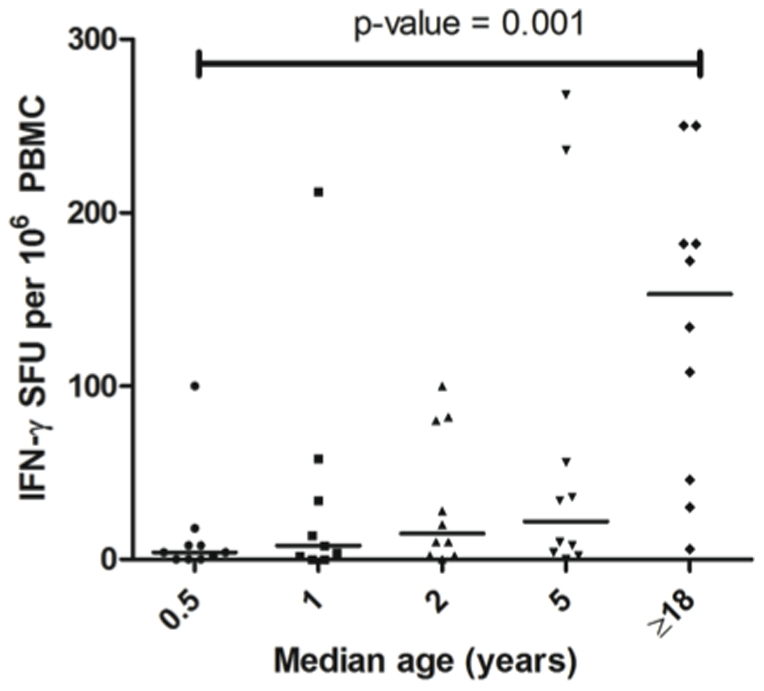
Magnitude of peripheral blood mononuclear cell IFN-γ responses to 3D7 MSP1_42_ according to age. The magnitude of response was measured by counting the spot forming units (SFU) for PBMCs stimulated with MSP1_42_ after subtracting SFU for PBMCs incubated with culture medium/PBS (background range 0–50 SFUs/10^6^ PBMCs). There was an age-related increase in the median magnitude of IFN-γ responses (*P* = 0.0065, Kruskall-Wallis test) with adults ≥18 years having higher frequencies than 0.5 year olds (*P* = 0.001, Dunn's *post hoc* test). X-axis is median age in years and Y-axis is IFN-γ SFU per 10^6^ PBMCs.

Due to the limited blood volume obtainable from young children, we conducted time-course experiments comparing 24, 48, 60 and 72 hour incubation periods (data not shown). These condition-optimization experiments demonstrated that a 60 hour incubation period resulted in measureable IFN-γ recall responses to MSP-1_42_ without resulting in appreciable cell death or inducing high background *in vitro*, and therefore was selected as the optimal incubation period for ELISPOT assays used in this study. The proportion of IFN-γ ELISPOT responders to MSP1_42_ did not significantly change from age 0.5 to 5 years, but increased from 20% for infants with median age 0.5 years to 90% for adults ≥18 years (*P* = 0.01, Table 2). However, significant increases with age were observed for the magnitude of IFN-γ responses, with a median of 5 to 155 SFU/10^6^ PBMCs across age groups (*P* = 0.001, Fig. 1). PBMCs from all 49 individuals examined generated IFN-γ in response to SEB super antigen (data not shown), confirming that all age groups were immune competent by this criterion.

**Table 2:** Proportion of individuals by age group with IFN-γ responses to MSP1_42_-3D7 as detected by ELISPOT.

Age Group (years)	Number IFN-γ Responders/Number Tested (%)
0.5	2/10 (20)*
1	4/9 (44)
2	5/10 (50)
5	5/10 (50)
≥18	9/10 (90)*

Notes: The proportion of responders did not significantly differ when comparing children less than 5 years old across different age groups (*P*>0.05).

*However by two-tailed Fisher's exact test infants 0.5 years of age had significantly fewer responders compared to adults older than 18 years (*P* = 0.01).

### *Ex vivo* resting CD4 and CD8 T cell effector memory subsets differ by age

**Figure 2:**
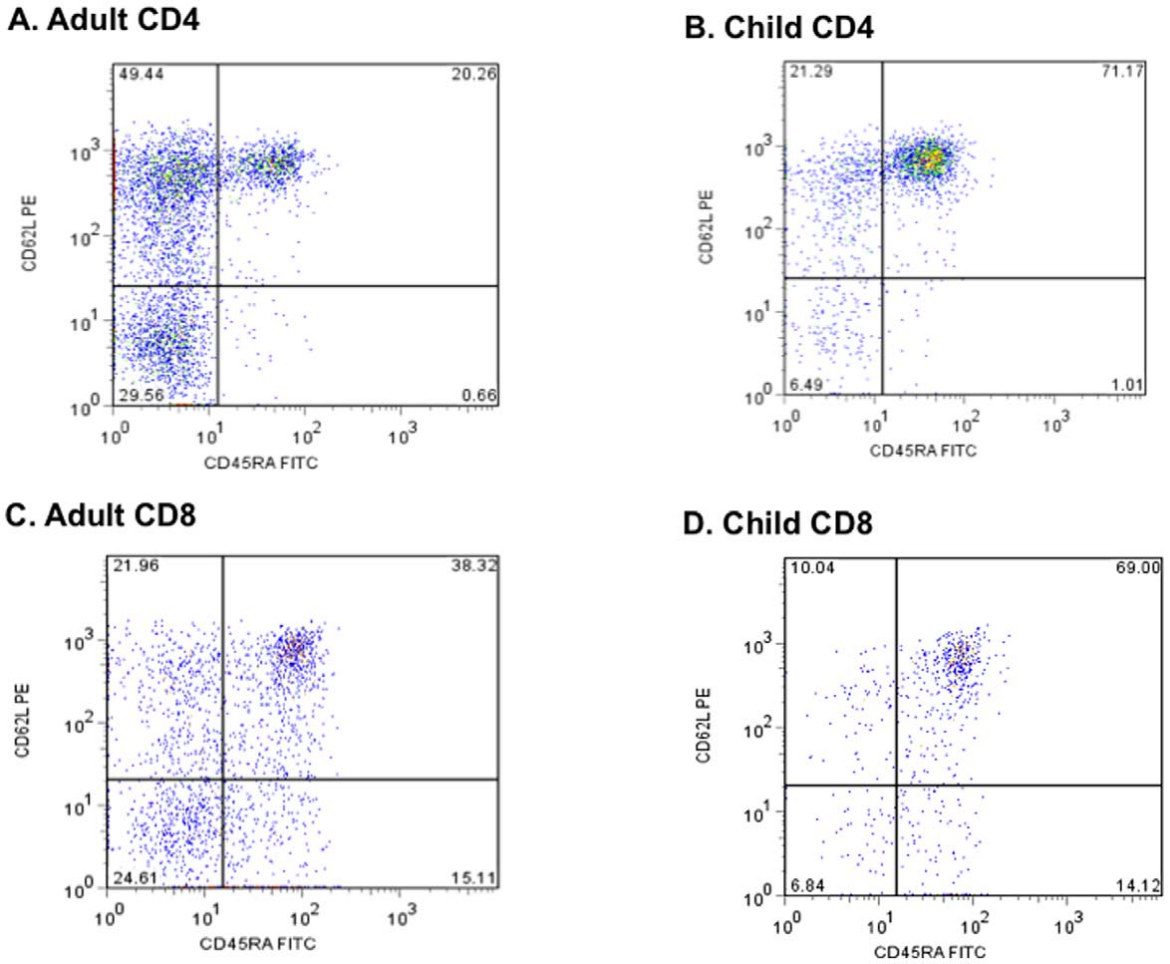
Representative dot plots showing the proportions of CD4 and CD8 T cells subsets defined by CD45RA and CD62L surface expression in adults and children. Dot plot (**A**) Adult CD4, (**B**) Child CD4, (**C**) Adult CD8, (**D**) Child CD8 T cell subsets.

**Figure 3:**
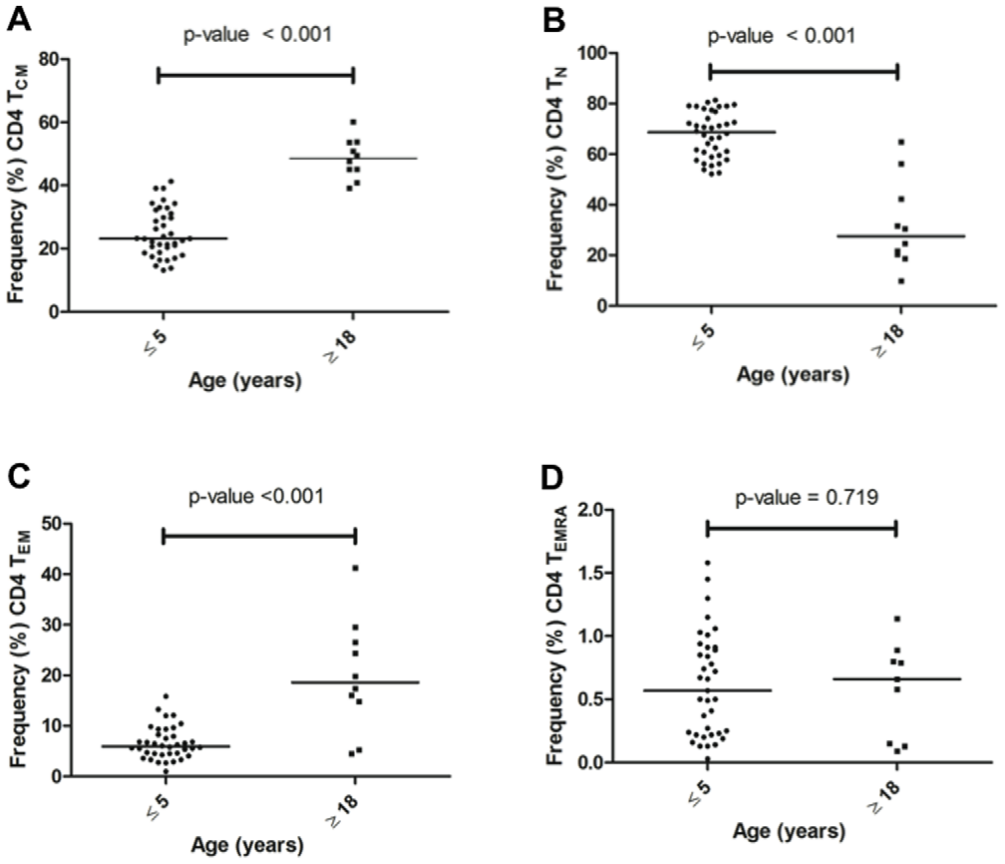
Proportion of resting CD4 T cell subsets across age groups. The frequency of each T cell subset was compared between adults (≥18 years old) and children (≤5 years old) for (**A**) Central memory, T_CM_: CD4^+^ CD45RA^−^ CD62L^+^; (**B**) naïve T cells, T_N_: CD4^+^ CD45RA^+^ CD62L^+^; (**C**) effector memory, T_EM_: CD4^+^ CD45RA^−^ CD62L^−^; and (**D**) RA-expressing effector memory, T_EMRA_: CD4^+^ CD45RA^+^ CD62L^−^. The median frequency of CD4^+^ T_CM_ and T_EM_ was significantly lower for children compared to adults (*P*<0.001, Mann Whitney test). Whereas the median frequency of CD4^+^ T_N_ was significantly higher for children compared with adults (*P*<0.001). The median frequency of CD4^+^ T_EMRA_ was similar for the two age groups (*P* = 0.719).

The proportions of various CD4 T-cell memory subsets were determined for freshly isolated PBMCs prior to *in vitro* exposure to MSP1_42_. Representative dot plots for PBMCs from an adult and a 1 year old child are shown in Fig. 2A and 2B. Overall, adults (n = 10) had a higher frequency of CD4 T_CM_ (CD45RA^−^ CD62L^+^) and T_EM_ (CD45RA^−^ CD62L^−^) compared to children ≤5 years (n = 39, *P*<0.001; Fig. 3A and 3C). In contrast, children had a higher frequency of T_N_ (CD45RA^+^ CD62L^+^) than adults (*P*<0.001, Fig. 3B); whereas the proportion of CD4 T_EMRA_ (CD45RA^+^ CD62L^−^) was <1%, and similar across age groups (*P* = 0.719, Fig. 3D).

**Figure 4:**
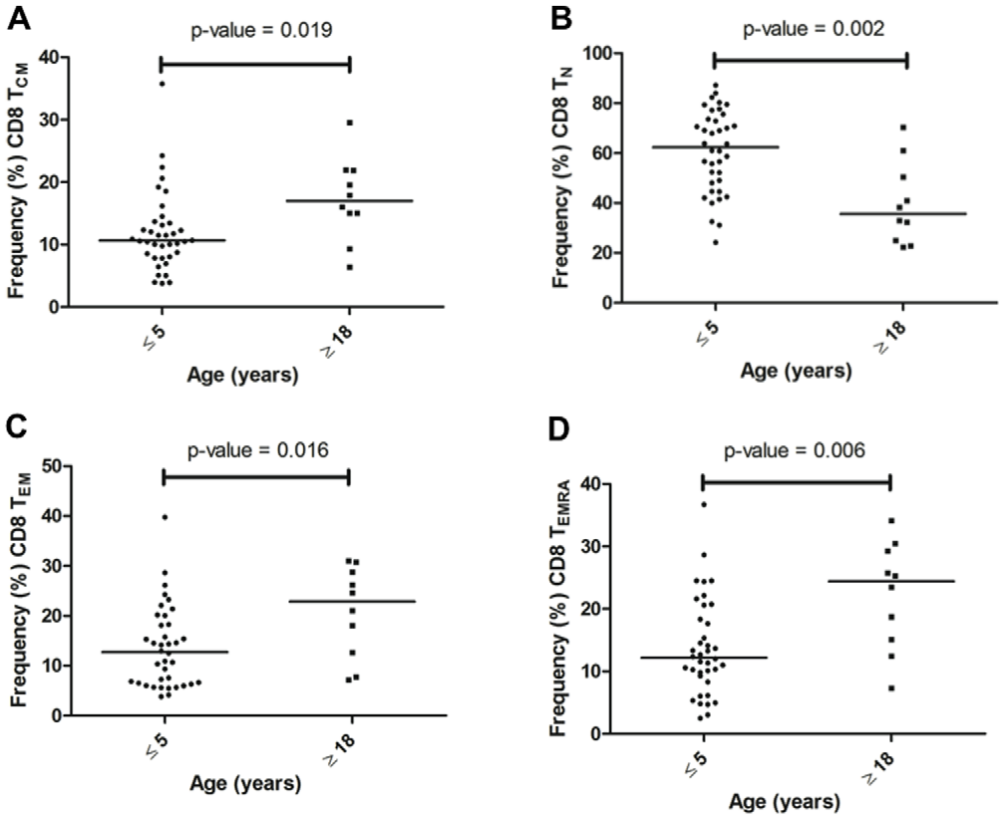
Proportion of resting CD8 T cell subsets across age groups. The frequency of each T cell subset was compared between adults (≥18 years old) and children (≤5 years old) for (**A**) Central memory, T_CM_: CD8^+^ CD45RA^−^ CD62L^+^; (**B**) naïve T cells, T_N_: CD8^+^ CD45RA^+^ CD62L^+^; (**C**) effector memory, T_EM_: CD8^+^ CD45RA^−^ CD62L^−^; and (**D**) RA-expressing effector memory, T_EMRA_: CD8^+^ CD45RA^+^ CD62L^−^. The median frequency of CD8^+^ T_CM_, T_EM_ and T_EMRA_ were significantly lower for children compared to adults (*P* = 0.019, *P* = 0.016 and *P* = 0.006, respectively, Mann Whitney test). In contrast, the median frequency of CD8^+^ T_N_ was significantly higher for children compared with adults (*P* = 0.0002).

PBMCs were stained in parallel for CD8 T-cell memory subsets as described above. This approach was necessary because a 4-color flow cytometer was the only instrument available to our laboratory in Kenya at the time the studies were performed. Figure 2C and 2D show representative histograms of the distribution of freshly isolated CD8 T-cell subsets from an adult and a 1-year old child. The age-related distribution of CD8 T-cell memory subsets was similar to that of CD4 cells, with T_CM_ (Fig. 4A) and T_EM_ (Fig. 4C) frequencies being higher among adults compared to children (*P* = 0.019 and 0.016, respectively) and the frequency of CD8 T_N_ (Fig. 4B) being higher in children compared to adults (*P* = 0.002). However, unlike the case for CD4 T_EMRA_ cells that showed no difference in frequency by age, CD8 T_EMRA_ cells (Fig. 4D) were readily detectable in both age groups, and were significantly higher among adults than children (24.4% and 12.2%, respectively, *P* = 0.006).

### MSP1_42_ driven IFN-γ production CD4 and CD8 effector memory subsets differ by age

**Figure 5:**
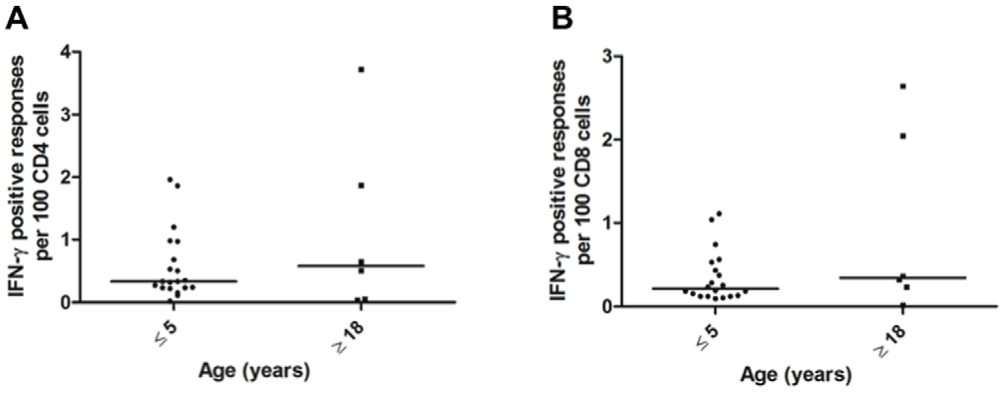
MSP1-specific IFN-γ responses generated from both CD^+^4 and CD8^+^ T cells. IFN-γ responses to MSP1_42_ (3D7 strain) were determined *ex vivo* by intracellular staining and flow cytometric analysis of cell surface marker expression. The frequency of IFN-γ positive responses per 100 CD4^+^ T cells (**A**) and CD8^+^ T cells (**B**) after *ex vivo* rMSP1_42_ stimulation were compared between children (≤5 years) and adults (≥18 years). There were no significant differences in the median number of IFN-γ responses generated by either CD4^+^ or CD8^+^ T cells by age group.

**Figure 6:**
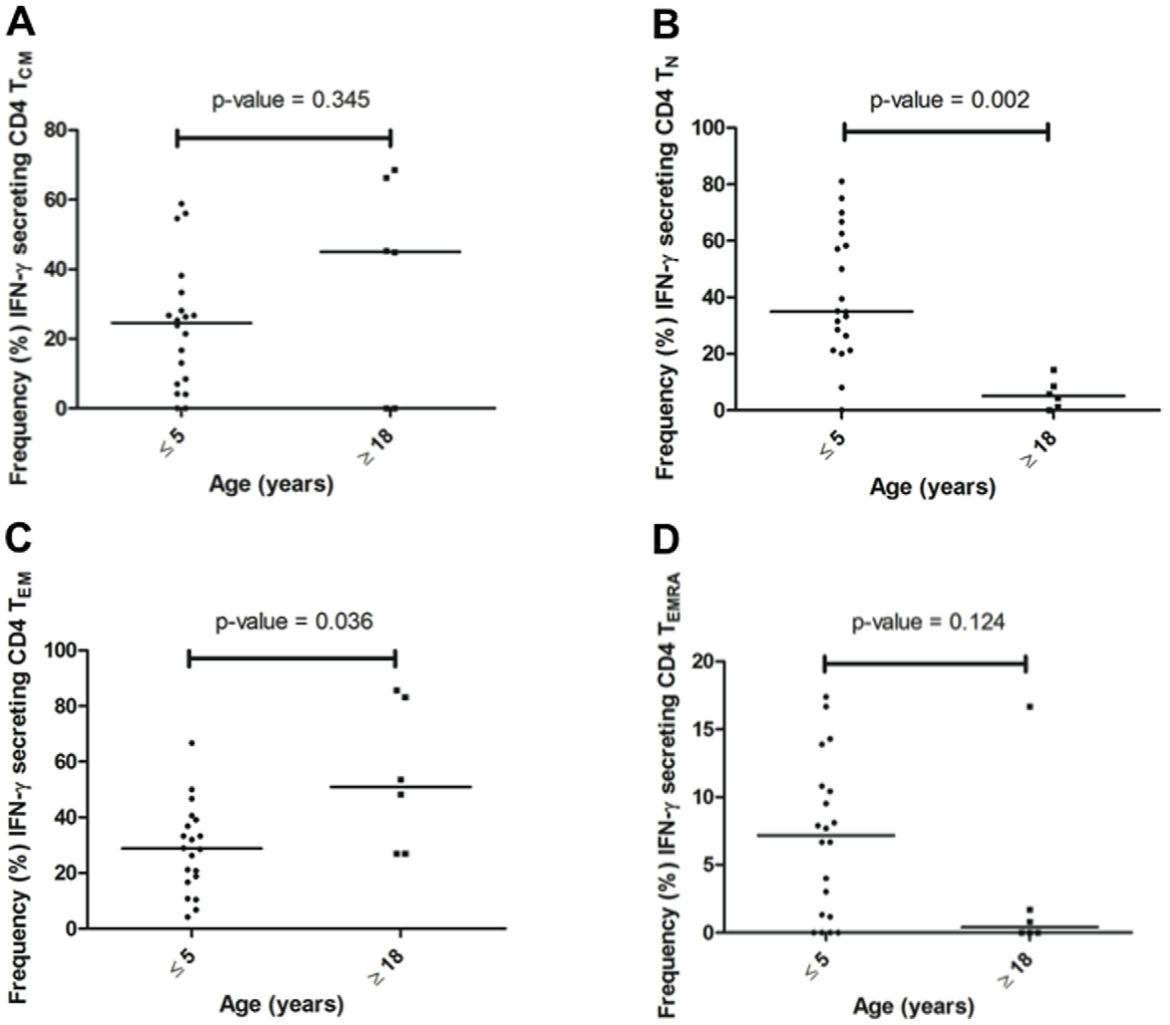
Proportion of MSP1_42_-specific IFN-γ secreting CD4+ T cell subsets across age groups. As described above, IFN-γ responses to MSP1_42_ (3D7 strain) were determined *ex vivo* by intracellular staining and flow cytometric analysis of cell surface marker expression. T cell subsets were defined by expression of CD45RA and CD62L surface markers: (**A**) T_CM_ (CD4^+^, CD45RA^−^, CD62L^+^); (**B**) T_N_ (CD4^+^, CD45RA^+^, CD62L^+^); (**C**) T_EM_ (CD4^+^, CD45RA^−^, CD62L^−^); and (**D**) T_EMRA_ (CD4^+^, CD45RA^+^, CD62L^−^). The phenotype of IFN-γ expressing CD4^+^ T cells in adults were predominantly T_CM_ and T_EM_ compared to the children who had IFN-γ expressing CD4^+^ T cells generated from all four subsets including those phenotypically T_EMRA_ and T_N_. Significant differences (Mann Whitney test) in median frequencies were observed for CD4^+^ T_EM_ subsets, which were more prevalent (*P*-value = 0.036) and CD4^+^ T_N_, which were less prevalent (*P* = 0.002) in adults compared to children. Differences observed in the frequencies of CD4^+^ T_CM_ and T_EMRA_ between age groups did not reach significance due to small sample size.

MSP1_42_-specific CD4 and CD8 T-cells were detected after *in vitro* stimulation followed by intracellular staining for IFN-γ and flow cytometric analysis, as described above. The median frequency (%) of MSP1_42_ driven CD4 and CD8 cells that stained positive for intracellular IFN-γ was less than 1% (ranging from nil to ∼3.8%), and did not differ significantly according to age (Fig. 5A and 5B). In order to determine the phenotype of the T-cell producing IFN-γ in response to MSP1_42_ stimulation, IFN-γ positive CD4 and CD8 T-cells were identified by flow cytometry and then back-gated into the memory T-cell subsets defined by CD45RA and CD62L expression. This analysis revealed that both adults and children had MSP1-specific IFN-γ producing CD4 T_CM_ (Fig. 6A) with median frequencies of 45.0% and 24.5%, respectively (*P* = 0.345). These differences were not significant due to the wide range of responses within each age group (from nil to ∼75% of all IFN-γ positive CD4 cells within the gate). However, the frequency of IFN-γ^+^ CD4 T_EM_ was significantly greater among adults than children (50.9% vs. 28.8%, Fig. 6C, *P* = 0.036). In contrast, the median frequency of MSP1_42_ driven IFN-γ^+^ CD4 T_N_ (Fig. 6B) was higher in children compared to adults (34.9% and 5.1%, respectively, *P* = 0.002). The median frequency of MSP1_42_-specific IFN-γ^+^ CD4 T_EMRA_ (Fig. 6D) was not significantly different among children and adults, 7.2% and 0.4%, respectively.

**Figure 7:**
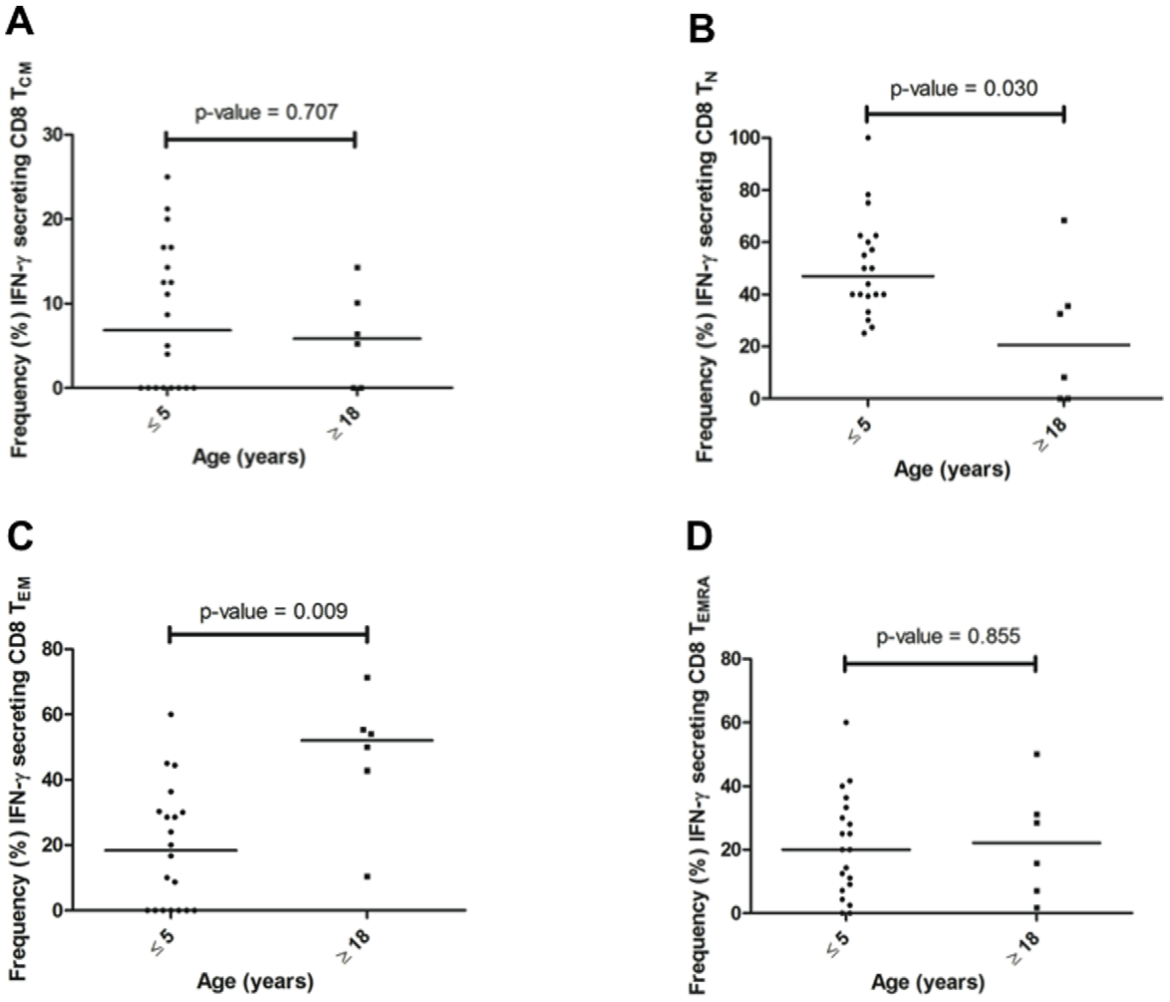
Proportion of MSP1_42_-specific IFN-γ secreting CD8+ T cell subsets across age groups. IFN-γ responses to MSP1_42_ (3D7 strain) were determined *ex vivo* by intracellular staining and flow cytometric analysis of cell surface marker expression. T cell subsets were defined by expression of CD45RA and CD62L surface markers: (**A**) T_CM_ (CD8^+^, CD45RA^−^, CD62L^+^); (**B**) T_N_ (CD8^+^, CD45RA^+^, CD62L^+^); (**C**) T_EM_ (CD8^+^, CD45RA^−^, CD62L^−^); and (**D**) T_EMRA_ (CD8^+^, CD45RA^+^, CD62L^−^). The predominant IFN-γ expressing CD8^+^ T cell in adults displayed a T_EM_ phenotype and this was significantly different than children (*P* = 0.009) who, similar to CD4^+^ T cells, had IFN-γ expression in response to MSP1_42_ from all four CD8^+^ T cell subsets with a predominance of phenotypically naïve secreting T cells (*P* = 0.030) compared to adults. IFN-γ responses observed for CD8^+^ T_CM_ and T_EMRA_ were of similarly low frequencies across age groups.

Even though classical endogenous MHC class I-restricted antigen processing and presentation may be considered unlikely for parasites that reside in erythrocytes, CD8 T-cells specific to blood stage antigens have been described in mouse malaria models [29], [30]. Therefore, we also performed studies to detect MSP1_42_-specific IFN-γ expressing CD8 T-cells and their effector/memory phenotypes. Of the CD8 T-cells that produced IFN-γ, the frequencies of T_CM_ (Fig. 7A) and T_EMRA_ (Fig. 7D) were similar among children and adults (*P* = 0.707 and 0.855, respectively). In contrast, the median frequency of T_EM_ cells (Fig. 7C) was significantly higher among adults compared to children (52.1% and 18.3%, respectively, *P* = 0.009) while CD8 T_N_ (Fig. 7B) were significantly more frequent among children than adults (47.0% and 20.5%. respectively, *P* = 0.030). The median frequency of MSP1_42_-specific IFN-γ^+^ CD8 T-cell responses mirrored the overall age-dependent shift in total CD8 T-cell effector memory subsets independent of antigen specificity (as shown in Fig. 4).

### The proportion of CD4 and CD8 T-cell subsets differs by age

In order to visualize the relative contributions of CD4 and CD8 T-cells subsets to the resting pool of T cells as well as to MSP1_42_-specific IFN-γ responses present in children ≤5 years of age and adults, proportions of each cell type were compared.

**Figure 8:**
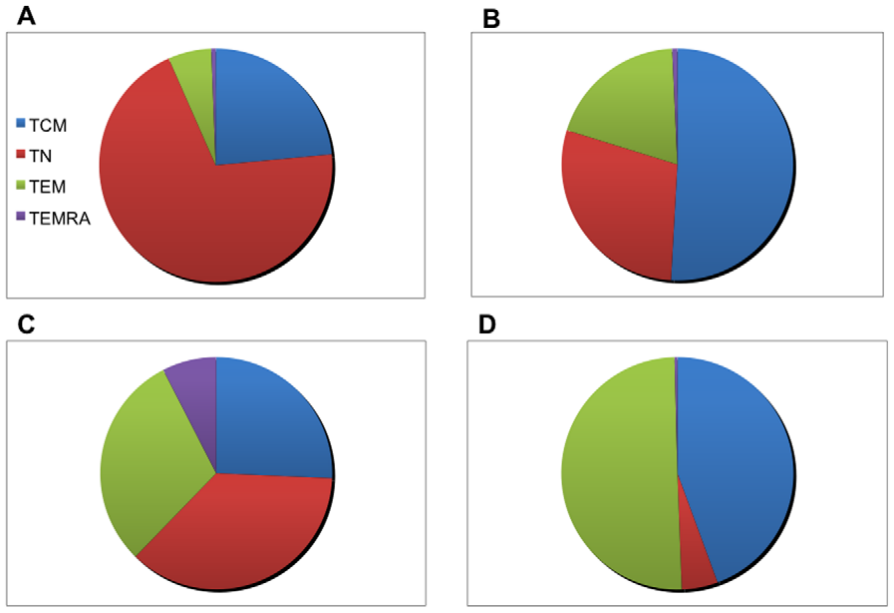
Proportion of resting and MSP1-specific IFN-γ producing CD4 T cell subset in children compared to adults. Top left panel (A) shows the proportion of resting CD4 T cell subsets for children ≤5 years old (from Fig. 3) with the lower left panel (C) showing the proportion of IFN-γ producing CD4 T cell subsets in response to rMSP1_42_ stimulation (from Fig. 6). Top right panel (B) shows the proportion of resting CD4 T cell subsets for adults ≥18 years old (from Fig. 3) with the lower right panel (D) showing the proportion of IFN-γ producing CD4 T cell subsets in response to rMSP1_42_ stimulation (from Fig. 6).

**Figure 9:**
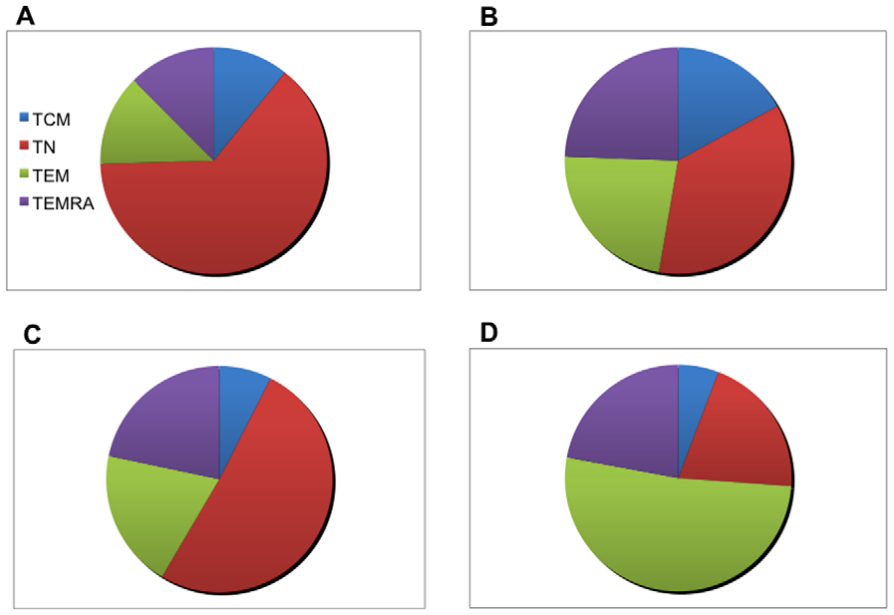
Proportion of resting and MSP1-specific IFN-γ producing CD8 T cell subset in children compared to adults. Top left panel (A) shows the proportion of resting CD8 T cell subsets for children ≤5 years old (from Fig. 4) with the lower left panel (C) showing the proportion of IFN-γ producing CD8 T cell subsets in response to rMSP1_42_ stimulation (from Fig. 7). Top right panel (B) shows the proportion of resting CD8 T cell subsets for adults ≥18 years old (from Fig. 4) with the lower right panel (D) showing the proportion of IFN-γ producing CD8 T cell subsets in response to rMSP1_42_ stimulation (from Fig. 7).

Figure 8 reveals that children have predominantly (68.7%) CD4 naïve-like, transitional CD4 T-cells (T_N_) cells compared to adults who have more (48.6%) CD4 T_CM_ cells in their resting pool. Even though children produce the same level of IFN-γ in response to stimulation with rMSP1_42_ as shown in Figure 5, the CD4 T cell subset responsible for this function in order of rank for children is T_N_ (34.9%)>T_EM_ (28.8%)>T_CM_ (24.5%)>T_EMRA_ (7.2%), in contrast to IFN-γ producing CD4 T cell subsets in adults which rank from highest to lowest median frequency: T_EM_ (50.9%)>T_CM_ (45.0%)>T_N_ (5.1%)>T_EMRA_ (0.4%). Figure 9 demonstrates similar age-dependent differences in CD8 T-cell subset proportions. Children have predominantly (62.8%) CD8 T_N_ cells compared to adults who have significantly fewer (35.6%) CD8 T_N_ cells (*P*-value = 0.002). Again, children appeared able to produce similar levels of IFN-γ as adults in response to stimulation with rMSP1_42_ as shown in Figure 5, but the CD8 T-cell subset contributing to this function differs in order of rank for children with median frequencies for T_N_ (47.0%)>T_EM_ (20.0%)>T_EMRA_ (18.3%)>T_CM_ (6.9%), in contrast to IFN-γ producing CD8 T-cell subsets in adults which rank T_EM_ (52.1%)>T_EMRA_ (22.2%)>T_N_ (20.5%)>T_CM_ (5.8%).

## Discussion

Our study demonstrated profound qualitative differences in T-cell memory responses to an essential merozoite invasion ligand and antigen protein, MSP1_42_, that correlate with age-related naturally acquired malaria immunity in residents of a holoendemic area of western Kenya. Although our study is limited by the small samples size, results show that the overall frequency of antigen-specific CD4 and CD8 T-cells that produced IFN-γ in response to MSP1_42_ were low and did not appear to differ according to parasitemia or ages ranging from 0.5 to 5 years and ≥18 years but there was a shift in hierarchy among various T-cell subsets responsive to MSP1_42_ such that the T_EM_ subset was the dominant cell type in adults in contrast to children who had more phenotypically naïve-like, T_N_ cells. These shifts were observed not only for IFN-γ producing CD4 cells, which might be anticipated based on the presumed importance of CD4 helper T-cells in generating and maintaining antibody mediated responses by B-cells, but also for CD8 T-cells, which are thought to be important in generating immunity to pre-erythrocytic rather than blood stage malaria antigens [31], [32]. Categorizing samples based on the presence of parasitemia or parasite density did not appear to influence IFN-γ responses T-cell subset dominance, however most of our study participants were aparasitemic and asymptomatic. Furthermore, the observed patterns of CD4 T-cell responses did not correlate with antibody responses to MSP1_42_ but there was weak correlation between CD8 responses and IgG responses to MSP1_42_. The potential importance for both CD4 and CD8 T-cells in determining resistance to blood stage parasitemia has been suggested by experimental infection of mice with *P. chabaudi* [33]. Absence of parasitemia in adults and differential parasite density in children suggest that different levels of immunity exist within this population and could be affected by parasite, human and external factors such as intensity of repeated malaria exposure. Longitudinal cohort studies looking at how age, repeated parasite exposure and clinical episodes influence the development of T-cell immunity to multiple antigens are underway. In addition, malaria-specific T-cell responses may differ in those residing in hypoendemic areas who are exposed to malaria antigens intermittently and with less intensity.

The overall magnitude of MSP1_42_ driven IFN-γ responses observed for CD4 and CD8 T-cells was generally weaker than that reported for recent studies of regulatory T cell responses in Gambian children with severe and mild malaria [34], [35] and malaria naïve volunteers challenged experimentally a single time with *P. falciparum* infected erythrocytes [36]. One likely explanation for this difference is that that the current study used a single, defined malaria antigen to stimulate T-cell recall responses, whereas the above mentioned studies and other reports used schizont extracts or infected erythrocytes as stimuli. Schizont extracts and infected erythrocytes contain not only MSP1 (presumably the non-processed pre-protein as well as various processed fragments) but also multiple other merozoite antigens and parasite moieties that may stimulate innate as well adaptive responses by T-cells and IFN-γ secreting NK cells [34], [37]. IFN-γ production by non-T cell types were not ascertained within this study but would explain the differences in frequencies observed between adults and children by ELISPOT (Fig. 1) that were not reflected in the T-cell specific ICS results (Fig. 5) that did not appear to differ by age. The frequencies reported here are consistent with IFN-γ secreting peripheral blood and cord blood mononuclear cells in our previously published studies using recombinant MSP1_33_, MSP1_42_ and peptide epitopes contained within MSP1_33_ to study T-cell IFN-γ recall responses in adults as well as newborns in Kenya [38], [39]. These earlier studies also established that the 3D7 strain of malaria is among the most common alleles of the T-cell epitope containing MSP1_33_ fragment circulating in western Kenya [38].

To determine the *ex vivo* frequency of phenotypically distinct T-cell populations, we compared freshly isolated CD4 and CD8 cell effector-memory subsets defined by CD45RA and CD62L [25]. As expected, there was a significantly greater proportion of T_CM_ and T_EM_ CD4 cell subsets among adults compared to children, whereas most CD4 cells were T_N_ in children relative to adults. This is consistent with other studies that compared the proportion of T cell subsets in older children and adults and observed a similar maturation pattern associated with age [40], [41]. There were very few terminally differentiated (T_EMRA_) CD4 cells present in these healthy individuals regardless of age. Similarly, the proportion of CD8 T_CM_ and T_EM_ cells increased with age and formed the majority of the CD8 T-cell compartment in adults as compared to more naïve-like (T_N_) CD8 T-cells found in young children. In contrast to CD4 T_EMRA_ that represented less than 1% of the total CD4 cell pool (Fig. 3), the frequency of T_EMRA_ within the CD8 cell pool increased from ∼15% in children 5 years and younger to over 20% in adults (Fig. 4). The observed age-related increase in the population of mature memory T-cell phenotype could be explained by the fact that as individuals age, they are exposed to a myriad of microbial antigens hence expanding the memory pool to these pathogens. When comparing the proportion of *ex vivo* T-cell subsets to the proportion of MSP1-specific IFN-γ producing T-cell subsets, in general the IFN-γ producing T-cell subsets reflect the proportion of *ex vivo* T-cell subsets present in each age group. For example, children have more T_N_ cells compared to adults who have relatively more T_CM_ and T_EM_ – and not surprisingly this distribution of T-cell subsets is reflected by the MSP-1 specific IFN-γ producing T-cell subsets. The exception to this ‘proportional-distribution by age’ is the CD4 T_EMRA_ cells, which do not differ between adults and children *ex vivo* but are over represented as a source of IFN-γ production in response to MSP-1 in the children but not the adults. The picture is slightly different for the CD8 T-cell subsets, where the proportion of *ex vivo* T_CM_ and T_EMRA_ are higher for adults compared to children, but the MSP-1 specific T_CM_ and T_EMRA_ making IFN-γ appear the same across age groups, with the dominant cell type making IFN-γ in the children being the T_N_ cells in contrast to the T_EM_ for the adults. Future studies will reveal if this particular CD8 T-cell subset is associated with fewer high-density infections over time and/or fewer episodes of clinical malaria. To date, no studies have compared the relative contributions from CD4 and CD8 T-cell effector subsets (i.e., T_EM_ versus T_EMRA_) in protection from asexual parasitemia or clinical infections.

The necessity of T-cell maturation and differentiation for the acquisition of malaria immunity is further supported by changes in the quality of IFN-γ recall responses to MSP1_42_ stimulation across age groups as presented here. IFN-γ has been associated with resolution of *Plasmodium* infection in humans and animal models [42], [43], and synergizes with other cytokines and nitric oxide to eliminate parasites [44]. An age-related increase in malaria-specific IFN-γ recall responses has been observed in several other studies of individuals from malaria endemic areas [45]. Children in Africa and Papua New Guinea were more efficient producers of IFN-γ to sporozoite or merozoite antigens, suggesting an association between antigen-specific IFN-γ production and reduced pathology [42]. Further, fewer CD4 IFN-γ producing cells in Gabonese children with acute malaria were associated with hyperparasitemia [46] and higher prevalence and magnitude of IFN-γ were observed at the end of rainy season (transmission season) than dry season in a seasonally endemic area of Gambia [47]. Taken together, these studies suggest that IFN-γ is a biomarker of recent exposure and/or clinical immunity to malaria. It is known that long term cultures may skew antigen specific responses, but our observation that children consistently secreted IFN-γ from naïve T-cells while adults had mature memory T-cells as the primary source of the cytokines argues against selective survival of a specific subset by the culture condition. The differential secretion of IFN-γ by CD4 and CD8 T-cells in adults and children (Figs. 6 and 7) has implications for the sustainability of this essential cytokine response.

Lack of sterile immunity for those residing in malaria endemic areas and waning of semi-protective immunity observed in residents of malaria endemic regions who travel to non-malarial zones [48] have suggested that long lasting immunologic memory to *P. falciparum* is not possible under natural exposure conditions. Cytokine recall responses could therefore be attributed to immunologic ‘boosting’ by repeat infections that may be derived from short-lived T-cells [49] or possibly other IFN-γ producing cells such as NK cells or monocytes. Nevertheless, adults returning to endemic areas appear to re-acquire clinical immunity more rapidly than children [50], [51] suggesting that some form of long-term memory to malaria was established. An alternate explanation is that a mature immune system acquires antigen-specific effector memory and/or central memory T-cells more efficiently than an immature immune system. An infant's ability to develop immunologic memory may thus be impaired due to age-inherent differences in antigen recognition, presentation and dendritic cell function [52], [53].

Historically demonstration of immunologic memory to malaria has been supported primarily by observations from serologic studies [54], [55], [56]. However, there is a growing consensus that antibody levels may be a more accurate measurement of cumulative malaria exposure history as opposed to the sole means by which protection is conferred [57]. Though it is well accepted that memory T-cells are an important component for protection against a variety of infectious pathogens [50], [51], there is limited information on human memory T-cells specific to malaria [45], [49]. The nature of memory T-cell populations elicited in malaria-naïve North Americans vaccinated with the 3D7 and FVO strains of MSP1_42_ demonstrated induction of antigen-specific memory CD4 T-cells [22], however the contribution of CD8 T-cell memory subsets was not evaluated. Studies on individuals residing in *P. vivax* malaria endemic areas have shown an increased proportion of memory CD8 T-cells subsets [52], supporting the premise that T-cell maturity is as important as antibodies in the development of protection against malaria. Although we cannot conclusively determine the origin of the blood stage specific CD8 T-cells within this study, it is clear that CD8 T-cells play a crucial function by secreting IFN-γ, a cytokine that has been shown to be important in control of malaria parasites. It has been demonstrated in mouse models of malaria that CD8 T-cells are able to ‘see’ malaria blood stage antigens through the process of cross-presentation [58], thus it is possible this mechanism is also important in human immunity to malaria.

Advances in polychromatic flow cytometry have demonstrated the heterogeneity of T-cell immunity and characterized ‘atypical’ antigen-specific memory T-cell populations [12]. Thus, our unexpected observation that IFN-γ was generated from phenotypically naïve-like T cells (T_N_) in response to MSP1_42_ from children, but not adults, could be an indication that malaria is driving an effector function from a population of atypical “transitional” T-cells. Since these cells have not undergone full maturation, there is high likelihood of effector T_N_ attaining a state of anergy and, thus leading to failure to contain infection. It is not known whether antigen experienced T_N_ differentiate into T_EM_ or T_CM_, become anergic or undergo apoptosis. Given that infants born to *P. falciparum* infected mothers may experience malaria attacks earlier than those whose mothers do not have malaria during pregnancy [55], it will be important to incorporate into longitudinal cohort studies the impact of fetal antigenic sensitization on malaria-specific T- cell differentiation during early childhood.

## Materials and Methods

### Ethics Statement

Ethical approvals were obtained from the University Hospitals of Cleveland Institutional Review Board for Human Studies at Case Western Reserve University and the Ethical Review Committee at the Kenya Medical Research Institute. Informed, written consent was obtained from each adult study participant and from the parent or guardian of minor study participants prior to enrollment in this study.

### Study population and study area

This was an age-structured study involving 49 participants grouped according to median age of 0.5, 1, 2, 5 and ≥18 years. All participants were healthy lifelong residents of a malaria holoendemic region of Nyanza Province in western Kenya. The study was conducted in the region near the Chulaimbo Rural Health Training Center managed by the Kenya Ministry of Health. Insecticide treated bed nets were not widely distributed in the area at the time the study was conducted (February and March 2007). Historically, the entomological inoculation rate in this area has been estimated at 0.65 to 0.79 infectious bites per person per day [54]. None of the study participants had a history of clinical malaria or taken anti-malarial drugs within the previous 4 weeks. Signed informed consent was obtained from adults, defined as residents who were ≥18 years old, and from the parent or guardian of participants who were 0.5 to 5 years old.

### Blood collection and PBMC isolation

Approximately 2–5 ml and 8–10 ml venous blood samples were collected into heparin anti-coagulated tubes from 0.5–5 and ≥18 year olds, respectively. Total white blood cell counts (WBC) and differentials describing proportions of lymphocytes, monocytes and polymorphonuclear leukocytes were assessed using a Coulter Counter (Coulter AcT Diff 2, Beckman Coulter, Miami, FL). The absolute number of CD4 and CD8 cells per µl of whole blood was determined according to WBC and flow cytometry using anti-CD4 and anti-CD8 antibodies. Absolute numbers of lymphocytes per µl blood were obtained from the Coulter Counter and the percentages of CD3, CD4, and CD8 cells within the lymphocyte population were acquired from flow cytometry data. The percentages of CD3, CD4, and CD8 cells were matched to the absolute number of lymphocytes to calculate the absolute numbers of these T-cell subsets. PBMC's were separated from fresh whole blood by Ficoll-hypaque density gradient centrifugation and suspended in culture medium (RPMI 1640 (GIBCO, Invitrogen, Paisley, Scotland UK) supplemented with 10% heat inactivated human AB serum, 50 µg/ml gentamicin, 10 mM HEPES and 2 mM L-glutamine). PBMCs were used at a concentration of 5×10^5^/well for IFN-γ ELISPOT and T cell memory subset phenotyping.

### Light microscopy to detect *P. falciparum* infection

Thick and thin smears were prepared from venous blood samples at the same time PBMCs were obtained. The slides were air dried, fixed in 100% methanol and stained with 5% Giemsa for enumeration of *P. falciparum* infected erythrocytes. A smear was deemed negative when microscopic inspection showed no parasites after counting fields that included at least 200 leukocytes. Density of parasitemia was expressed as the number of asexual *P. falciparum*/µl blood assuming a leukocyte count of 8,000/µl of whole blood.

### Superantigens and malaria antigens

PBMCs were incubated with culture medium and PBS (blank control), Staphylococcal Enterotoxin B (SEB) at 2 µg/ml and recombinant MSP-1_42_ (3D7 allele) at 5 µg/mL [56]. SEB served as the positive control. Recombinant MSP-1_42_ was provided by Carole Long and Sanjay Singh (NIAID, NIH, Rockville MD). The construct contains T cell and B cell epitopes in the MSP1_33_ and MSP1_19_ fragments, respectively [57].

### Cell surface staining for *ex vivo* T-cell memory phenotypes

Five hundred thousand PBMCs were suspended in 100 µl 0.5% BSA-PBS (wash buffer) and stained with the following panel of antibodies to characterize the memory T cell phenotype: CD3-APC, CD4-PerCP or CD8- PerCP, Cy7, CD45RA -FITC and CD62L-PE. All antibodies were purchased from BD Biosciences and used according to the manufacturer's instructions. Stained cells were incubated in the dark at room temperature for 30 minutes. Labeled cells were washed with 2 ml wash buffer and fixed with 500 µl 4% paraformaldehyde for 15 min at 4°C in the dark. At least 10,000 gated events were acquired using a FACSCalibur™ flow cytometer (Becton-Dickinson).

### IFN-γ enzyme linked immunospot (ELISPOT)

MultiScreen 96-well plates (Millipore, Bellirica MA) were coated overnight at 4°C with capture anti-human IFN-γ antibody (Endogen M-700A, Worcester MA) at a final concentration of 5 µg/ml. The next day, the plates were washed 3 times with PBS and blocked with 100 µl 10% fetal calf serum for 2 hours at room temperature. Plates were then washed 3 times with PBS, and 5×10^5^ cells seeded per well. SEB and MSP1_42_ were added in respective wells and cultured for 60 hours in a humidified incubator with 5% CO_2_ at 37°C. The plates were subsequently washed with PBS and secondary anti-human IFN-γ antibody (Endogen M-700B) added at a final concentration of 0.75 µg/ml culture medium followed by incubation for 90 minutes at 37°C. Plates were washed 3 times with PBS-Tween, horseradish peroxidase (HRP) conjugated streptavidin (DAKO P0397, Carpinteria CA) added at a 1∶2000 dilution, and incubated for 2 hours at room temperature. Finally, the plates were washed 3 times with PBS and 1% 3-amino-9-ethyl-carbazole in 0.1 M acetate buffer (HRP substrate) was added to visualize spots. Plates were scanned and spot forming units (SFU) counted by Immunospot satellite analyzer (Cellular Technology, Cleveland OH). An individual was defined as an IFN-γ responder to MSP1_42_ if the frequency of SFUs per 10^6^ PBMCs in the stimulated well was significantly greater than the well containing culture medium alone using Fisher's exact test. The range of IFN-γ secreting cells in the unstimulated wells was 0–7 SFU/million PBMC.

### Long term culture and intracellular cytokine and surface staining for T cell memory subsets

At the same time that IFN-γ ELISPOT assays were started, parallel aliquots of PBMCs from all 49 donors were seeded at a concentration of 2.5×10^5^ cells/200 µl culture medium in duplicate wells of 96-well round bottom culture plates, washed 60 hours later and re-suspended in fresh culture medium supplemented with 20 U recombinant human IL-2. All 25 PBMC samples that had positive IFN-γ ELISPOT responses (determined after 60 hours *ex vivo* incubation as described above) and those that had enough cells but lacked detectable IFN-γ responses were further evaluated for T cell memory subsets in the long-term cultures supplemented with IL-2. On day 6, from the initial seeding, cells from duplicate wells were pooled, washed and incubated for 18 hours with fresh culture medium alone, SEB or MSP1_42_. During the last 6 hours of culture, brefeldin A was added to allow for intracellular accumulation of IFN-γ. Cells were washed with 20 mM EDTA-PBS and transferred into 5 ml polystyrene tubes, washed with 0.5% BSA-PBS and labeled for detection of cell surface CD3, CD4, CD8, CD45RA, and CD62L at room temperature for 30 minutes. Fixing was done with 4% paraformaldehyde for 15 min at 4°C in the dark. Cells were then washed twice with HEPES plus 0.1% saponin (permeabilization buffer) and cell membranes permeabilized for 30 min at 4°C in the dark. 50 µL permeabilization buffer and anti-human IFN-γ APC antibody were then added and incubation continued for 30 minutes. Finally, cells were washed twice with 500 µl permeabilization buffer, resuspended in 0.5 ml BSA-PBS and 5×10^4^ gated events per tube acquired for flow cytometric analysis. For IFN-γ ICS, 50,000 gated lymphocyte events were acquired and any IFN-γ spots from the unstimulated cells were subtracted from the MSP1 stimulated cells for each study participant to determine frequency of MSP1-specific IFN-γ producing cells per age group as shown in Figure 5. The range of IFN-γ positive cells found in the unstimulated CD4 and CD8 stained samples were 0.03–1.56% and 0.05–0.74%, respectively. For Figures 6 and 7 the proportion of MSP1-speicific IFN-γ producing cells in each of the four T cell subsets (CD45 RA v CD62L) was then determined.

### Enzyme linked immunosorbent assay (ELISA) for detection of total IgG antibodies

IgG antibodies were measured by ELISA. Recombinant MSP142 protein was dissolved in 0.01 M phosphate-buffered saline (PBS) to a concentration of 0.1 ug/ml and added to immulon-4 plates (Dynex Technologies, Chantily, VA). After overnight incubation at 40C, washing and blocking in 5% non-fat powdered milk in PBS, duplicate 50 ml samples of serum diluted 1/100 in 5% powdered milk were added to wells, and incubation was continued for 2 hours at room temperature. After washing, 50 ml of alkaline phosphatase-conjugated goat anti-human IgG (Jackson ImmunoResearch, West Grove, PA), diluted 1/1000 in 5% powdered milk was added and removed after 1 hour. The plate was washed three times and substrate p-nitrophenyl phosphate was added in accordance with the manufacturer's instructions (Sigma-Aldrich, St. Louis, MO). The reaction was stopped with 3N NaoH and optical density (OD) was measured at 405 nm. An antibody response was considered positive if it was three standard deviations above the mean of the malaria-naïve negative controls [59].

### Data analysis

Flow cytometric data were processed and analyzed using FlowJo software version 7.2 (Tree Star, San Carlos CA). Figure S1 is a representative histogram depicting the gating strategy used to capture MSP1_42_-specific IFN-γ expressing CD4+ and CD8+ T cells. Figure S2 shows the gating strategy to determine the proportions of effector-memory CD4 and CD8 T cell subsets expressing CD45RA and CD62L. There was no minimum event number required for a positive response since the proportion of each T-cell subset back-gated from IFN-γ producing cells (either CD4 or CD8, respectively) was reported for each individual (Figs. 6 and 7). The median frequency of each subset was used to demonstrate the phenotypic differences for the MSP1 IFN-γ producing T populations in children compared to adults (Figs. 8 and 9). Fisher's exact test was used to compare the proportion of IFN-γ responders across age groups and the difference between stimulated and unstimulated cells. Kruskal-Wallis test was used to compare the magnitude of IFN-γ secretion by ELISPOT and frequencies of T cell subsets across the five age categories. All analyses were done using the Graphpad program (Graphpad Prism™, La Jolla CA).

## Supporting Information

Figure S1 **Representative forward versus side scatter gating strategy for peripheral blood mononuclear cells from adult Kenyan study participant (panel A) examined for IFN-γ expression by CD4^+^ T cells incubated** ***ex vivo*** **for 7 days with culture medium with PBS (panel B) or recombinant MSP1_42_-3D7 (panel C) by FlowJo Software. Similar analyses were done in parallel for CD8+ T cells.**
(TIFF)

Figure S2 **Schematic representation from one individual showing the proportion of each T-cell subset based on the expression of the cell surface markers CD45RA and CD62L determined after gating for MSP1-specific IFN-γ expression by CD4^+^ T cells. Similar studies were done in parallel for CD8^+^ T cells.**
(TIFF)

## References

[1] Hogh B (1996). Clinical and parasitological studies on immunity to Plasmodium falciparum malaria in children.. Scand J Infect Dis.

[2] Taylor-Robinson AW (2002). A model of development of acquired immunity to malaria in humans living under endemic conditions.. Med Hypotheses.

[3] Artavanis-Tsakonas K, Tongren JE, Riley EM (2003). The war between the malaria parasite and the immune system: immunity, immunoregulation and immunopathology.. Clin Exp Immunol.

[4] Leri O, Perinelli P, Losi T, Mastropasqua M, Peri C (1997). [Malaria: recent immunological acquisitions and therapeutic prospects].. Clin Ter.

[5] Cohen S, Mc GI, Carrington S (1961). Gamma-globulin and acquired immunity to human malaria.. Nature.

[6] Langhorne J, Ndungu FM, Sponaas AM, Marsh K (2008). Immunity to malaria: more questions than answers.. Nat Immunol.

[7] Pombo DJ, Lawrence G, Hirunpetcharat C, Rzepczyk C, Bryden M (2002). Immunity to malaria after administration of ultra-low doses of red cells infected with Plasmodium falciparum.. Lancet.

[8] Edstein MD, Kotecka BM, Anderson KL, Pombo DJ, Kyle DE (2005). Lengthy antimalarial activity of atovaquone in human plasma following atovaquone-proguanil administration.. Antimicrob Agents Chemother.

[9] Egan A, Waterfall M, Pinder M, Holder A, Riley E (1997). Characterization of human T- and B-cell epitopes in the C terminus of Plasmodium falciparum merozoite surface protein 1: evidence for poor T-cell recognition of polypeptides with numerous disulfide bonds.. Infect Immun.

[10] Garraud O, Diouf A, Holm I, Perraut R, Longacre S (1999). Immune responses to Plasmodium falciparum-merozoite surface protein 1 (MSP1) antigen, II. Induction of parasite-specific immunoglobulin G in unsensitized human B cells after in vitro T-cell priming with MSP119.. Immunology.

[11] Schluns KS, Lefrancois L (2003). Cytokine control of memory T-cell development and survival.. Nat Rev Immunol.

[12] Chattopadhyay PK, Hogerkorp CM, Roederer M (2008). A chromatic explosion: the development and future of multiparameter flow cytometry.. Immunology.

[13] Huaman MC, Martin LB, Malkin E, Narum DL, Miller LH (2008). Ex vivo cytokine and memory T cell responses to the 42-kDa fragment of Plasmodium falciparum merozoite surface protein-1 in vaccinated volunteers.. J Immunol.

[14] Lee EA, Palmer DR, Flanagan KL, Reece WH, Odhiambo K (2002). Induction of T helper type 1 and 2 responses to 19-kilodalton merozoite surface protein 1 in vaccinated healthy volunteers and adults naturally exposed to malaria.. Infect Immun.

[15] Sakai T, Hisaeda H, Nakano Y, Zhang M, Takashima M (2003). Gene gun-based co-immunization of merozoite surface protein-1 cDNA with IL-12 expression plasmid confers protection against lethal Plasmodium yoelii in A/J mice.. Vaccine.

[16] Sallusto F, Lenig D, Forster R, Lipp M, Lanzavecchia A (1999). Two subsets of memory T lymphocytes with distinct homing potentials and effector functions.. Nature.

[17] Huaman MC, Mullen GE, Long CA, Mahanty S (2009). Plasmodium falciparum apical membrane antigen 1 vaccine elicits multifunctional CD4 cytokine-producing and memory T cells.. Vaccine.

[18] Erkeller-Yuksel FM, Deneys V, Yuksel B, Hannet I, Hulstaert F (1992). Age-related changes in human blood lymphocyte subpopulations.. J Pediatr.

[19] Hulstaert F, Hannet I, Deneys V, Munhyeshuli V, Reichert T (1994). Age-related changes in human blood lymphocyte subpopulations. II. Varying kinetics of percentage and absolute count measurements.. Clin Immunol Immunopathol.

[20] Sinigaglia F, Guttinger M, Kilgus J, Doran DM, Matile H (1988). A malaria T-cell epitope recognized in association with most mouse and human MHC class II molecules.. Nature.

[21] Lundie RJ, de Koning-Ward TF, Davey GM, Nie CQ, Hansen DS (2008). Blood-stage Plasmodium infection induces CD8+ T lymphocytes to parasite-expressed antigens, largely regulated by CD8alpha+ dendritic cells.. Proc Natl Acad Sci U S A.

[22] Wilson NS, Behrens GM, Lundie RJ, Smith CM, Waithman J (2006). Systemic activation of dendritic cells by Toll-like receptor ligands or malaria infection impairs cross-presentation and antiviral immunity.. Nat Immunol.

[23] Todryk SM, Bejon P, Mwangi T, Plebanski M, Urban B (2008). Correlation of memory T cell responses against TRAP with protection from clinical malaria, and CD4 CD25 high T cells with susceptibility in Kenyans.. PLoS One.

[24] Todryk SM, Walther M, Bejon P, Hutchings C, Thompson FM (2009). Multiple functions of human T cells generated by experimental malaria challenge.. Eur J Immunol.

[25] Podoba JE, Stevenson MM (1991). CD4+ and CD8+ T lymphocytes both contribute to acquired immunity to blood-stage Plasmodium chabaudi AS.. Infect Immun.

[26] Horowitz A, Newman KC, Evans JH, Korbel DS, Davis DM (2010). Cross-talk between T cells and NK cells generates rapid effector responses to Plasmodium falciparum-infected erythrocytes.. J Immunol.

[27] McCall MB, Roestenberg M, Ploemen I, Teirlinck A, Hopman J (2010). Memory-like IFN-gamma response by NK cells following malaria infection reveals the crucial role of T cells in NK cell activation by P. falciparum.. Eur J Immunol.

[28] Walther M, Jeffries D, Finney OC, Njie M, Ebonyi A (2009). Distinct roles for FOXP3 and FOXP3 CD4 T cells in regulating cellular immunity to uncomplicated and severe Plasmodium falciparum malaria.. PLoS Pathog.

[29] Franklin BS, Parroche P, Ataide MA, Lauw F, Ropert C (2009). Malaria primes the innate immune response due to interferon-gamma induced enhancement of toll-like receptor expression and function.. Proc Natl Acad Sci U S A.

[30] Malhotra I, Wamachi AN, Mungai PL, Mzungu E, Koech D (2008). Fine specificity of neonatal lymphocytes to an abundant malaria blood-stage antigen: epitope mapping of Plasmodium falciparum MSP1(33).. J Immunol.

[31] Jackola DR, Ruger JK, Miller RA (1994). Age-associated changes in human T cell phenotype and function.. Aging (Milano).

[32] Spring MD, Chelimo K, Tisch DJ, Sumba PO, Rochford R (2010). Allele specificity of gamma interferon responses to the carboxyl-terminal region of Plasmodium falciparum merozoite surface protein 1 by Kenyan adults with naturally acquired immunity to malaria.. Infect Immun.

[33] Saule P, Trauet J, Dutriez V, Lekeux V, Dessaint JP (2006). Accumulation of memory T cells from childhood to old age: central and effector memory cells in CD4(+) versus effector memory and terminally differentiated memory cells in CD8(+) compartment.. Mech Ageing Dev.

[34] Luty AJ, Lell B, Schmidt-Ott R, Lehman LG, Luckner D (1999). Interferon-gamma responses are associated with resistance to reinfection with Plasmodium falciparum in young African children.. J Infect Dis.

[35] Taylor-Robinson AW, Phillips RS (1992). Functional characterization of protective CD4+ T-cell clones reactive to the murine malaria parasite Plasmodium chabaudi.. Immunology.

[36] Kern DE, Grabstein KH, Okuno K, Schreiber RD, Greenberg PD (1989). Identification of a unique T cell-derived lymphokine that primes macrophages for tumor cytotoxicity.. J Immunol.

[37] Moormann AM (2009). How might infant and paediatric immune responses influence malaria vaccine efficacy?. Parasite Immunol.

[38] Riley EM, Morris-Jones S, Blackman MJ, Greenwood BM, Holder AA (1993). A longitudinal study of naturally acquired cellular and humoral immune responses to a merozoite surface protein (MSP1) of Plasmodium falciparum in an area of seasonal malaria transmission.. Parasite Immunol.

[39] Winkler S, Willheim M, Baier K, Schmid D, Aichelburg A (1999). Frequency of cytokine-producing T cells in patients of different age groups with Plasmodium falciparum malaria.. J Infect Dis.

[40] Achtman AH, Bull PC, Stephens R, Langhorne J (2005). Longevity of the immune response and memory to blood-stage malaria infection.. Curr Top Microbiol Immunol.

[41] Baird JK (1998). Age-dependent characteristics of protection v. susceptibility to Plasmodium falciparum.. Ann Trop Med Parasitol.

[42] Bouchaud O, Cot M, Kony S, Durand R, Schiemann R (2005). Do African immigrants living in France have long-term malarial immunity?. Am J Trop Med Hyg.

[43] Deloron P, Chougnet C (1992). Is immunity to malaria really short-lived?. Parasitol Today.

[44] Siegrist CA (2007). The challenges of vaccine responses in early life: selected examples.. J Comp Pathol.

[45] Willems F, Vollstedt S, Suter M (2009). Phenotype and function of neonatal DC.. Eur J Immunol.

[46] Wang L, Crouch L, Richie TL, Nhan DH, Coppel RL (2003). Naturally acquired antibody responses to the components of the Plasmodium falciparum merozoite surface protein 1 complex.. Parasite Immunol.

[47] Woehlbier U, Epp C, Kauth CW, Lutz R, Long CA (2006). Analysis of antibodies directed against merozoite surface protein 1 of the human malaria parasite Plasmodium falciparum.. Infect Immun.

[48] Yazdani SS, Mukherjee P, Chauhan VS, Chitnis CE (2006). Immune responses to asexual blood-stages of malaria parasites.. Curr Mol Med.

[49] Corran P, Coleman P, Riley E, Drakeley C (2007). Serology: a robust indicator of malaria transmission intensity?. Trends Parasitol.

[50] Esser MT, Marchese RD, Kierstead LS, Tussey LG, Wang F (2003). Memory T cells and vaccines.. Vaccine.

[51] Tsuji T, Nibu R, Iwai K, Kanegane H, Yachie A (1994). Efficient induction of immunoglobulin production in neonatal naive B cells by memory CD4+ T cell subset expressing homing receptor L-selectin.. J Immunol.

[52] Jangpatarapongsa K, Sirichaisinthop J, Sattabongkot J, Cui L, Montgomery SM (2006). Memory T cells protect against Plasmodium vivax infection.. Microbes Infect.

[53] Precopio ML, Betts MR, Parrino J, Price DA, Gostick E (2007). Immunization with vaccinia virus induces polyfunctional and phenotypically distinctive CD8(+) T cell responses.. J Exp Med.

[54] Beier JC, Oster CN, Onyango FK, Bales JD, Sherwood JA (1994). Plasmodium falciparum incidence relative to entomologic inoculation rates at a site proposed for testing malaria vaccines in western Kenya.. Am J Trop Med Hyg.

[55] Schwarz NG, Adegnika AA, Breitling LP, Gabor J, Agnandji ST (2008). Placental malaria increases malaria risk in the first 30 months of life.. Clin Infect Dis.

[56] Singh S, Kennedy MC, Long CA, Saul AJ, Miller LH (2003). Biochemical and immunological characterization of bacterially expressed and refolded Plasmodium falciparum 42-kilodalton C-terminal merozoite surface protein 1.. Infect Immun.

[57] Udhayakumar V, Anyona D, Kariuki S, Shi YP, Bloland PB (1995). Identification of T and B cell epitopes recognized by humans in the C-terminal 42-kDa domain of the Plasmodium falciparum merozoite surface protein (MSP)-1.. J Immunol.

[58] Miyakoda M, Kimura D, Yuda M, Chinzei Y, Shibata Y (2008). Malaria-specific and nonspecific activation of CD8+ T cells during blood stage of Plasmodium berghei infection.. J Immunol.

[59] Noland GS, Hendel-Paterson B, Min XM, Moormann AM, Vulule JM (2008). Low prevalence of antibodies to preerythrocytic but not blood-stage Plasmodium falciparum antigens in an area of unstable malaria transmission compared to prevalence in an area of stable malaria transmission.. Infect Immun.

