# Supplementary figures and images for: Age-Related Differences in Naturally Acquired T Cell Memory to Plasmodium falciparum Merozoite Surface Protein 1
Source: PLoS One. 2011 Sep 16;6(9):e24852. doi: 10.1371/journal.pone.0024852 (PMC3174209; doi:10.1371/journal.pone.0024852)

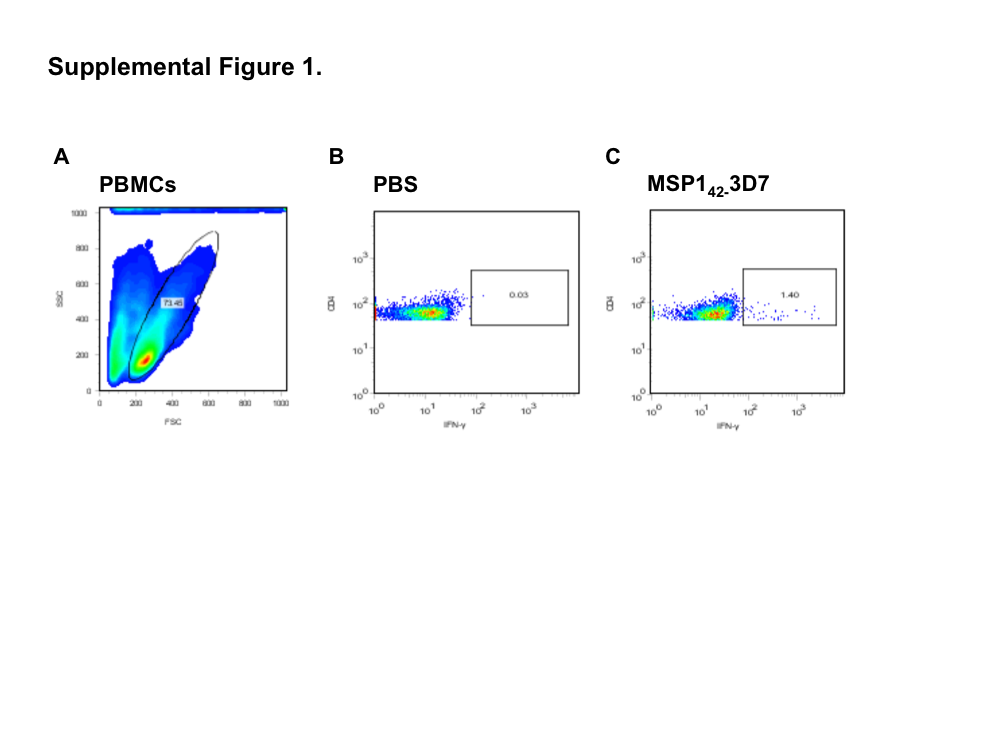

Supplement: Figure S1 — Representative forward versus side scatter gating strategy for peripheral blood mononuclear cells from adult Kenyan study participant (panel A) examined for IFN-γ expression by CD4+ T cells incubated ex vivo for 7 days with culture medium with PBS (panel B) or recombinant MSP142-3D7 (panel C) by FlowJo Software. Similar analyses were done in parallel for CD8+ T cells. (TIFF) [file pone.0024852.s001.tiff]

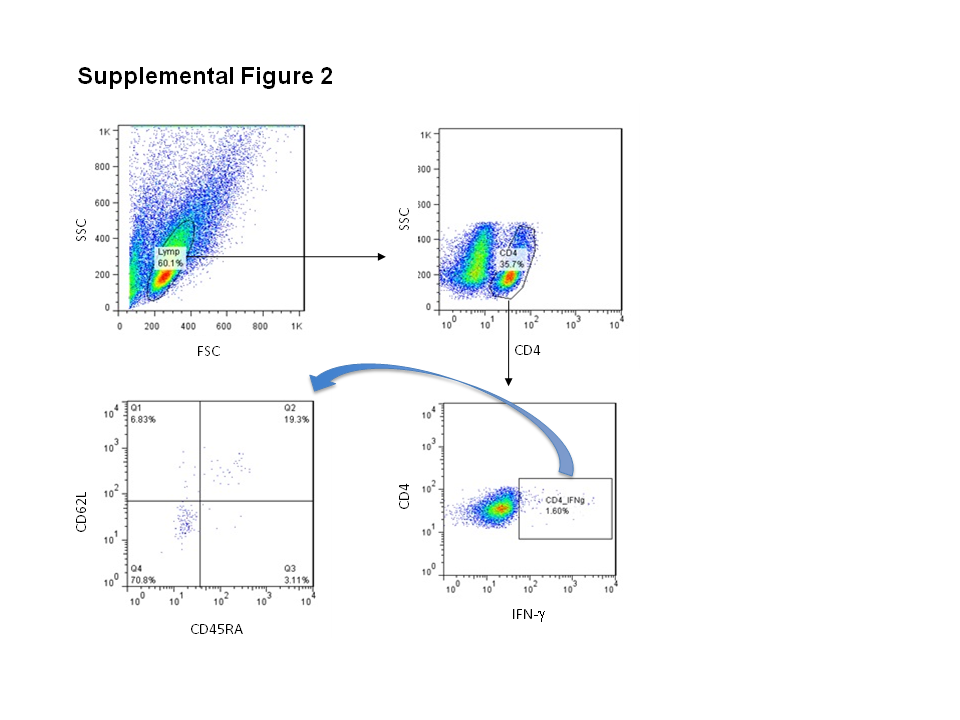

Supplement: Figure S2 — Schematic representation from one individual showing the proportion of each T-cell subset based on the expression of the cell surface markers CD45RA and CD62L determined after gating for MSP1-specific IFN-γ expression by CD4+ T cells. Similar studies were done in parallel for CD8+ T cells. (TIFF) [file pone.0024852.s002.tiff]
